# NGS-QCbox and Raspberry for Parallel, Automated and Rapid Quality Control Analysis of Large-Scale Next Generation Sequencing (Illumina) Data

**DOI:** 10.1371/journal.pone.0139868

**Published:** 2015-10-13

**Authors:** Mohan A. V. S. K. Katta, Aamir W. Khan, Dadakhalandar Doddamani, Mahendar Thudi, Rajeev K. Varshney

**Affiliations:** 1 International Crops Research Institute for the Semi-Arid Tropics (ICRISAT), Hyderabad, India; 2 School of Plant Biology and Institute of Agriculture, The University of Western Australia, Crawley, Australia; The University of Hong Kong, HONG KONG

## Abstract

Rapid popularity and adaptation of next generation sequencing (NGS) approaches have generated huge volumes of data. High throughput platforms like Illumina HiSeq produce terabytes of raw data that requires quick processing. Quality control of the data is an important component prior to the downstream analyses. To address these issues, we have developed a quality control pipeline, NGS-QCbox that scales up to process hundreds or thousands of samples. Raspberry is an in-house tool, developed in C language utilizing HTSlib (v1.2.1) (http://htslib.org), for computing read/base level statistics. It can be used as stand-alone application and can process both compressed and uncompressed FASTQ format files. NGS-QCbox integrates Raspberry with other open-source tools for alignment (Bowtie2), SNP calling (SAMtools) and other utilities (bedtools) towards analyzing raw NGS data at higher efficiency and in high-throughput manner. The pipeline implements batch processing of jobs using Bpipe (https://github.com/ssadedin/bpipe) in parallel and internally, a fine grained task parallelization utilizing OpenMP. It reports read and base statistics along with genome coverage and variants in a user friendly format. The pipeline developed presents a simple menu driven interface and can be used in either *quick* or *complete* mode. In addition, the pipeline in *quick* mode outperforms in speed against other similar existing QC pipeline/tools. The NGS-QCbox pipeline, Raspberry tool and associated scripts are made available at the URL https://github.com/CEG-ICRISAT/NGS-QCbox and https://github.com/CEG-ICRISAT/Raspberry for rapid quality control analysis of large-scale next generation sequencing (Illumina) data.

## Introduction

Next generation sequencing (NGS) technologies generates large volumes of data that are proven to be cost effective over conventional sequencing methods. Rapid decline in costs of the data generation in recent years has boosted rapid adoption of NGS based applications towards unraveling biological questions [1]. NGS approaches generate large volumes of data that are cost effective over conventional sequencing methods. Availability of genome wide information of species was a major constraint until NGS was introduced and adopted. The primary application of such studies involve *de novo* genome assembly, whole genome re-sequencing, targeted studies apart from other specialized analyses such as RNA-Seq. For example, several plant genomes have been sequenced [2] and now efforts are underway to harness the diversity for crop improvement though re-sequencing of thousands of germplasm lines for instance rice (http://www.gigasciencejournal.com/content/3/1/7), maize [3], sorghum [4], chickpea [5] have been sequenced. NGS technologies typically generate gigabytes to terabytes of raw data and in due course the data accumulates to the scale of terabytes to petabytes in public archives. For example, as of May 2015, the European Nucleotide Archive (ENA) contains a massive dataset of 13.7 trillion read sequences (1,757.3 trillion bases) with the number of reads deposited doubling every 22.9 months (http://www.ebi.ac.uk/ena/about/statistics#sra_growth). Notably, in the period between 2006 and 2010, ENA has shown significant increase in the volume of data deposited and hence reflects the data generated. In addition to the data storage related issues, the challenge is to process and hence develop efficient tools to use the huge data towards downstream analysis in a limited time [6,7]. The data needs to be analyzed and archived for re-use at a later stage. Hence, prior to the downstream analysis, the NGS data typically needs to be processed for quality thereby generating high quality reads. Several tools like NGS QC Toolkit [8], FastQC (http://www.bioinformatics.babraham.ac.uk/projects/fastqc/) and HTSeq [9] exist for extracting high quality read data. But the existing tools/pipelines are capable of handling only few to tens of samples at an instance. Nevertheless, these tools could not address the issue of processing the huge volumes of data in parallel. Hence there is a pressing need for tools that can scale up to process thousands of samples simultaneously in short time. In this context, quality control (QC) of raw and large-scale NGS data demands automation.

In recent past, stand-alone quality control tools and pipelines have been developed to manage the overwhelming volume of data. For instance, quality control tools/pipelines like NGS QC Toolkit [8] (http://59.163.192.90:8080/ngsqctoolkit) and Python (http://www.python.org) based HTSeq [9] (http://www-huber.embl.de/users/anders/HTSeq/doc/overview.html) were developed to address these constraints but are slow. In general, these pipelines/tools are meant to work on datasets in serial manner that can be daunting for the end user while dealing with large datasets. Nowadays, not only the servers, but also modern personal computers include multicore processors and therefore several NGS tools have been developed to process the data in parallel by multi-threading. Keeping in view the requirement of an automated pipeline to analyze large-scale raw NGS data, a menu driven pipeline, namely NGS-QCbox that integrates Raspberry, an in-house developed tool, with other open source tools has been developed. The pipeline focuses on processing large datasets in parallel and provides informative and crisp statistics. Typically, service providers or labs involved in generating NGS data develop in-house scripts, for pre-processing of NGS data. NGS-QCbox aims to hasten and ease the processing of huge data in reasonable time frame.

The NGS-QCbox is meant to complement existing tools for QC. It aims to be a decision making tool in assisting the scientist to judge if sufficient quality data has been generated with an optimal coverage as the experiment demands.

## Results and Discussion

### Raspberry–a tool for FASTQ data statistics

The data generated from Illumina sequencing machines is in binary format (bcl). This is converted to FASTQ format, which we refer to as raw data. Depending on the number of samples or the data generated, often the raw data may be stored in compressed (gzip) or uncompressed format (plain text). In FASTQ format [10], each read is represented linearly as a record of four lines that includes identifier, sequence and its base quality information. The base quality information includes an offset of either 33 (HiSeq/MiSeq) or 64 (GAIIx). In order to assess the quality of the data generated, we have developed an in-house tool called Raspberry (v0.3) (https://github.com/CEG-ICRISAT/Raspberry) in C language utilizing HTSlib (v1.2) (http://htslib.org) towards computing read/base level metrics. The tool provides an account of total number of bases, reads, Q20 and Q30 bases, range of read length, average read length, range of quality, range of phred quality score, number of A/T/G/C and N characters and GC content. In addition, it provides a file with read lengths that could be plotted using a Python script, ‘read_length_distribution.py’ included in ‘utils’ folder of the package. Raspberry can be used as a stand-alone tool. It accepts compressed and uncompressed FASTQ format files as input. Raspberry, by default utilizes all the processors available on the machine. However, it allows user to change the number of processors to be used with the “–t” option. This could be beneficial if the server/workstation is under heavy load and the user has less number of processors allocated than the total available. This option would facilitate processing of the data in batches by the number of processors provided. Note that the number of processors opted translates to the number of samples processed at a given instance (batch). The datasets of legacy Illumina platforms that were encoded with a phred offset ‘64’ could be processed using the ‘–p 64’ option. By default the value is set to 33 which is the latest standard phred offset followed on HiSeq and MiSeq platforms. The manual made available online lists these use cases.

### Integrated pipeline

A top-level Python script (NGSQCbox-v0.1.py) presents a menu driven interface for the required input data and spawns tasks in parallel for each sample using in-built shell scripts and Bpipe [11] configuration files. Internally, Bpipe (v0.9.8.6_beta_1) was used to integrate NGS tools such as Raspberry (v0.3) (https://github.com/CEG-ICRISAT/Raspberry), Sickle (v1.200) (https://github.com/najoshi/sickle), Bowtie 2 (v2.1.0) [12], SAMtools (v0.1.19+) [13] and bedtools (v2.17.0) [14]. Within Bpipe, each of the components of the pipeline is represented as re-usable tasks or blocks of code that may run in parallel to reduce computational run time–task oriented parallelism.

**Fig 1:**
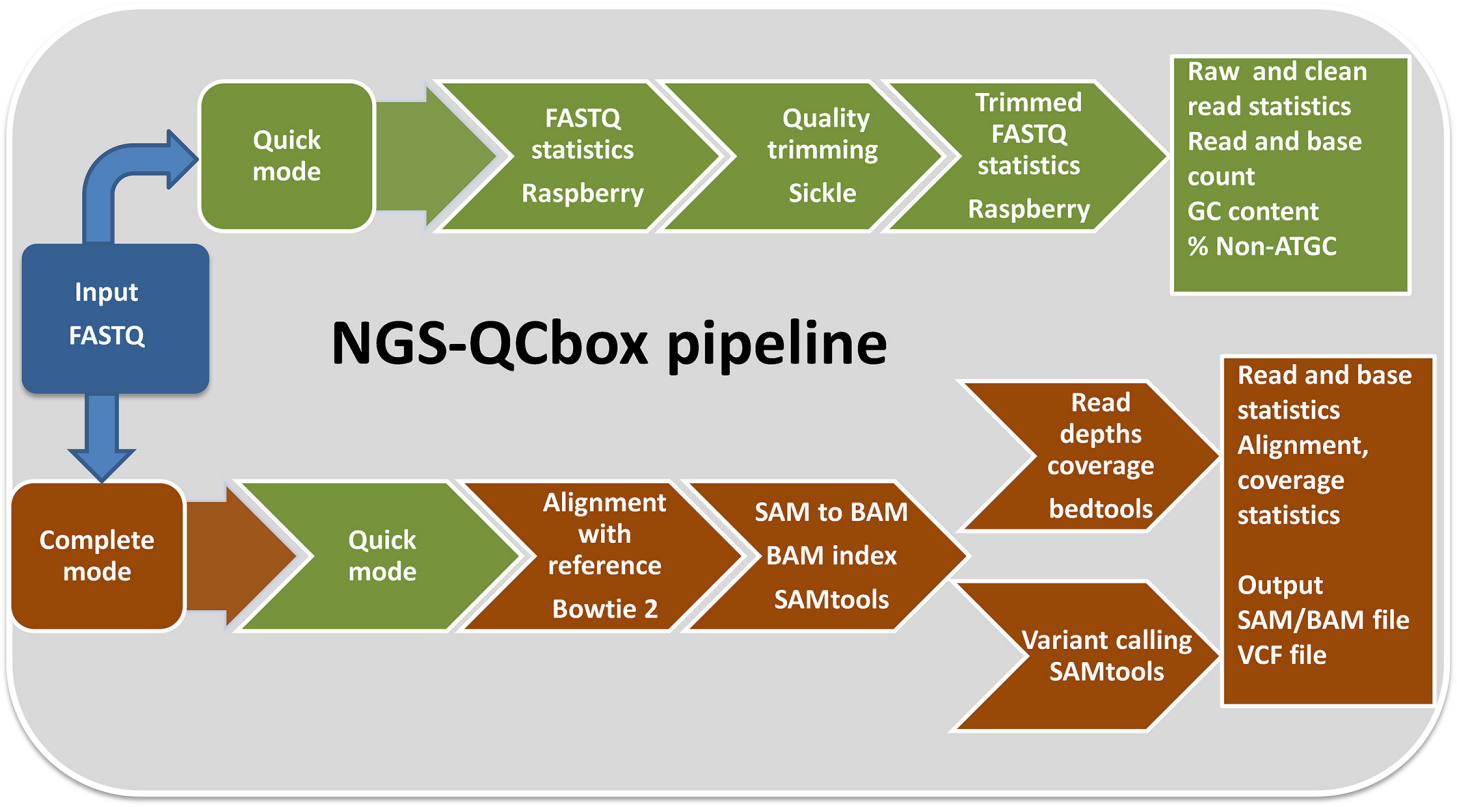
Flowchart of NGS-QCbox pipeline illustrating the two modes of usage namely *quick* and *complete*. NGS-QCbox comprises of two workflow modes namely *quick* and *complete*. In *quick* mode, read/base level metrics are computed in parallel using Raspberry, an in-house tool, both before and after quality trimming. On the other hand, *complete* mode is full-fledged quality control and variant calling pipeline that integrates quick mode and additionally generates genome coverage information in parallel. Quality of the data generated could be assessed using this information.

**Fig 2:**
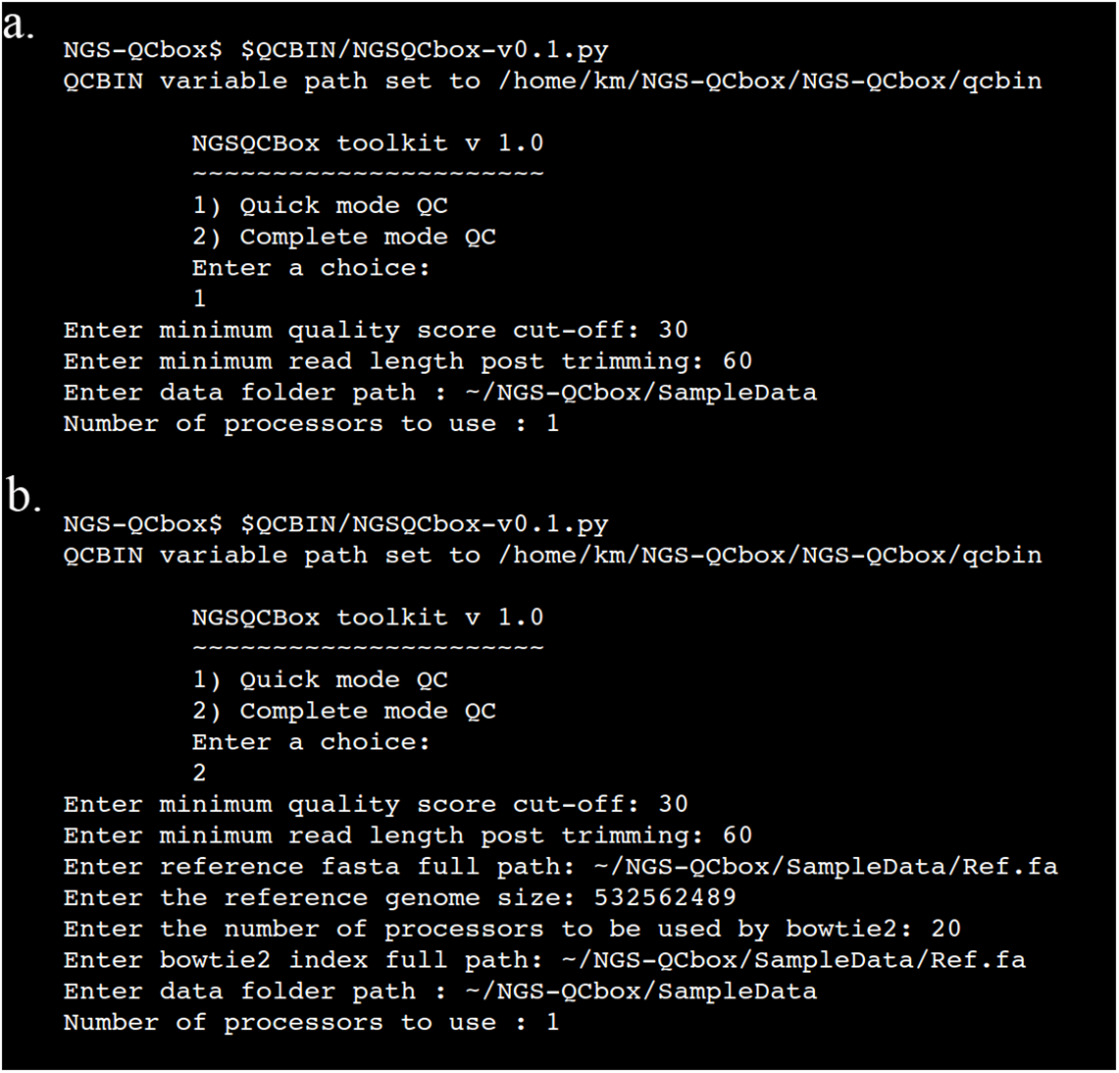
The menu driven interface for NGS-QCbox for quick and complete mode respectively. (a) Shows the prompt and the parameters such as cut-off phred score, minimum read length after trimming, data source and number of processors to be used for the quick QC mode. (b) Complete QC mode adds more parameters to quick mode like information related to the genome (bowtie index, genome size, number of processors used by bowtie).

The pipeline can be used in two modes: *quick* and *complete* (Fig 1). If the user is limited by time, *quick* mode could be used to have a general overview of the reads generated that includes base level metrics with quality trimming step. Alternately, it could be run in *complete* mode to generate additional information such as coverage, alignment, mean read depths and variants. The *complete* mode QC is in a way a full-fledged pipeline that covers processing raw reads to identifying variants. Fig 2 depicts the interface for the two modes of the pipeline. The parameters such as cut-off phred quality score, post trimming read length cut-off, data location and number of cores to be used for the quick QC mode. B) In addition to parameters included in quick QC mode, information related to the genome (bowtie index, genome size, number of processors used by bowtie) are incorporated as additional parameters in the complete QC mode.

Quality trimming by sickle includes parameters ‘-q 30’, ‘-l 50’, ‘-n’ and ‘-t sanger’, when run with default parameters. The alignment parameters of Bowtie 2 include ‘–end-to-end’ mode with user provided insert size information. The bedtools *genomecov* parameters include ‘-bga’ followed by the output processing using an in-house python script ‘genome_cov.py’ to compute the genome coverage from the alignment. Variant calling with SAMtools include the parameters ‘-uf’ to samtools *mpileup* and ‘-bvcg’ to bcftools respectively. In *quick* mode the user needs the sample information alone while in the *complete* mode one needs to provide additional information such as insert size of each sample size and Bowtie 2 index of the genome (see online manual). Users can freely modify the steps and subsequently the parameters involved by editing the bpipe scripts in the pipeline for both *quick* and *complete* mode QC.

### The workflow

The pipeline starts processing paired end reads using Raspberry. Base quality trimming of the paired end reads is performed using Sickle to produce high quality reads. Raspberry generates the metrics both before and after the base quality trimming step to help compare and assess the amount of reads/bases passing QC filter. This is essentially the quick QC mode. The *complete* QC mode includes the *quick* QC mode as well as the following steps. The high quality reads are aligned to the reference genome using Bowtie 2 to generate alignments in SAM/BAM format (https://samtools.github.io/hts-specs/SAMv1.pdf) followed by indexing with SAMtools. The singletons produced by the Sickle are also used in alignment. Bedtools is then used to compute the genome coverage based on the read depths at each base position. An in-house Python script, genome_cov.py (included with the tool source code) summarizes the output in terms of X coverage (1X, 2X, 5X, 10X, 15X). The X coverage trace could be used to evaluate the drop in read depth that may affect variant calling downstream. SAMtools is simultaneously used to call variants in VCF format (http://www.1000genomes.org/wiki/analysis/variant%20call%20format/vcf-variant-call-format-version-41) for each sample. The VCF files generated could be filtered and used for downstream applications such as GWAS, diversity analysis and development of markers for genotyping applications. The *complete* QC mode serves as a full-fledged pipeline for variant calling and is unique feature of the pipeline. An example file containing information on the output is illustrated in the S1 Table. Computation of genome coverage and variant calling steps use the same BAM files as input and hence these tasks have been parallelized internally to save time.

### Salient features of the pipeline

1. The pipeline accepts compressed/uncompressed paired-end Illumina FASTQ data.
2. Easy to use python based interface for fast (quick) and detailed (complete) processing of data.
3. Scalability–The pipeline is designed to process hundreds/thousands of samples in parallel (automated).
4. Batch processing of jobs–Even with the availability of limited number of processors all the samples can be processed in batch mode automatically.
5. Use of advanced shell features (Process substitution), task oriented parallelism, Python/multiprocessing, and HTSlib to gain performance in speed and save disk-space.
6. The pipeline computes and summarizes genome coverage and variant detection in parallel which reduces the processing time.
7. Available as docker image to ease portability of the pipeline.

### Benchmarking

The features of NGS-QCbox were compared with five well known tools/pipelines namely Prinseq-lite [15], NGS QC Toolkit, HTSeq, FastQC and FastX Toolkit (fastx_quality_stats) (http://hannonlab.cshl.edu/fastx_toolkit/index.html) (Table 1). The *quick* QC mode of NGS-QCbox was used for benchmarking. The unique features of the pipeline are simple menu interface, batch processing of jobs, task parallelization and information on genome coverage and variations.

**Table 1:** A comparative account of the features of NGS-QCbox pipeline with five similar pipeline/tools.

	NGS-QCbox	Prinseq-lite	NGS QC Toolkit	HTSeq	FastQC	FastX
Compressed FASTQ (input)	Y	N	N	N	N	N
Batch job processing	Y	N	N	N	Y/N	N
Genome coverage	Y	N	N	N	N	Y
Variations (SNP/INDEL)	Y	N	N	N	N	N
Menu interface	Y	N	N	N	Y	N
Feature richness	Y	Y	Y	N	Y	N
Task parallelization	Y	N	N	N	N	N

The symbols Y and N denote Yes and No respectively describing the presence or absence of the feature.

### Performance in processing large datasets

In order to test the scalability and the performance of NGS-QCbox, it was evaluated with data from 1, 100, 200 and 300 simulated paired-end samples (Table 2)**.** The tools were evaluated based on the performance observed with 1 processor against using 20 processors (parallel) with similar dataset size. The time consumed by NGS-QCbox to process one sample of size 4.38 Gb running on one processor was 217 seconds. This is a notable speedup of 2.76X over Prinseq-lite, 132X over NGS QC Toolkit, and 2.9X over HTSeq, 1.65X over FastQC and 4.9X over FastX. Similarly the time taken to process 100, 200 and 300 samples were in corresponding proportion because of the serial processing of the samples. In this case, with increase in the number of samples, all the programs scale up linearly with increase in data size (number of samples).

**Table 2:** Parallel performance comparison of NGS-QCbox with other tools/pipelines.

Quick QC mode							
Samples	Processors	NGS-QCbox	Prinseq-lite	NGS QC Toolkit	HTSeq	FastQC	FastX
		(seconds)	(seconds)	(seconds)	(seconds)	(seconds)	(seconds)
1	1	217	600	28,618	630	360	1,073
100	1	21,849	60,116	2,861,800*	63,513	36,121	107,300*
200	1	43,741	120,232*	5,523,600*	127,026*	72,318	214,600*
300	1	65,319	180,348*	8,285,400*	190,539*	108,477*	321,900*
1	20	217	NA	10,020	NA	NA	NA
100	20	3,472	NA	954,221	NA	NA	NA
200	20	4,636	NA	1,908,442*	NA	NA	NA
300	20	7,189	NA	2,862,663*	NA	NA	NA
**Complete QC mode**							
		**NGS-QCbox (seconds)**		**Shell script (sequential processing time in seconds)**			
1	1	2,800		2,838			
100	20	31,723		NA			

The tools were evaluated based on the performance observed with 1 processor against using 20 processors (parallel). To process one sample of size 4.38 Gb with one processor, NGS-QCbox consumes 217 seconds. This is a notable speedup of 2.76X over Prinseq-lite, 132X over NGS QC Toolkit, 2.9X over HTSeq, 1.65X over FastQC and 4.9X over FastX. In this case, with increase in the number of samples, all the programs scale linearly with increase in data size (samples). Similarly with 100, 200 and 300 samples, the speedups are the same order because of the serial processing of the samples. But when processing 100 samples in parallel with 20 processors the speedup obtained is 6.25X over the one processor run. Similar speedups of 9.4X and 9X were observed when comparing the runtime of 200 and 300 samples. This translates to the fact that the runtime to process each sample gets reduced to 23–34 seconds with parallelization which is a huge gain over running them serially. The “*” symbol indicates that the values are extrapolated based on the linear run time. Extrapolation is necessary because in such cases the run time exceeds a time period over days and months. NA indicates the program does not support parallelization. We have executed the flow of commands used in complete QC mode of pipeline into sequential order instead of parallel mode with one processor as input. It was observed that there was a loss of 38 seconds per sample when NGS-QCbox steps were ran sequentially. When complete QC mode was tested for 100 samples parallel processing gave a massive speedup of 8.82X.

On the other hand, NGS-QCbox runtime with 20 processors is the same 217 seconds as observed earlier with 1 processor (Table 2). This is because in both the cases (1 processor or 20 processors), threads are opened based on the number of samples (one sample per thread). Therefore if one sample is processed, it would use only one thread from the pool of threads. But the performance gain is evident with increasing the number of samples from 1 to 100, 200 and 300. While processing 100 samples in parallel with 20 processors, the speedup obtained is 6.29X over the one processor run. Better speedups of 9.4X and 9X were observed when comparing the runtime of 200 and 300 samples. This translates to the fact that the runtime to process each sample gets reduced to 23–34 seconds with parallelization which is a huge gain over running them serially. The programs such as Prinseq-lite, HTSeq and FastX run in serial, can only use one processor and hence were not considered for comparison in parallel. NGS-QCbox is much faster than NGS QC Toolkit when 20 processors were considered. In parallel mode (20 processors), NGS-QCbox ran 46X, 275X, 412X and 398X times faster than NGS QC Toolkit as evident from the comparisons of 1, 100, 200 and 300 samples respectively. However, sequential execution of the steps used in complete QC mode for a sample with one processor resulted in a loss of 38 seconds, underlining the efficiency of the pipeline in complete QC mode. Whereas scaling of number of samples to 100 with 20 processors gave a speedup of 8.82X compared to the run of one sample with one processor in complete QC mode (Table 2). In summary, parallelization drastically improved and facilitated processing of hundreds of samples. This would scale to thousands of samples but then disk I/O may be a constraint that might limit the performance. The mechanism of batch processing of samples in NGS-QCbox helps in containing the disk I/O because even though the number of samples exceeds the number of processors available, limited number of threads in a batch limit the disk read/writes proportionately and ensure that the disk I/O does not become a bottleneck while processing large number of samples. This would indirectly help contain the memory usage. NGS-QCbox consumes negligible amount of memory as it reads only a line at a time from a sample file and therefore is suitable for use on desktop machines.

NGS-QCbox can be used to process any paired end data from NGS experiments such as DNA-Seq, RAD-Seq, GBS, RNA-Seq. For processing single end read datasets one may need to use Raspberry independently.

The NGS-QCbox pipeline and Raspberry are available alternatively from dockerhub (https://registry.hub.docker.com) as a docker image (dadu/ngsqcbox:v0.2.1 for linux and dadu/ngsqcbox_win:v0.2 for Windows). Docker (docker.io) is a popular and portable lightweight linux container based technology to host applications in a virtual environment. This technology eases the process of distributing the application and thereby helps solve application related cross platform compatibility issues. The docker image solves the issue of dependencies across various linux platforms and Windows.

## Conclusions

NGS-QCbox is a generic pipeline that integrates open-source NGS tools in order to process large datasets of any organism. It was designed and implemented to take advantage of parallelization. Parallelization enables quick analysis of datasets that would otherwise be a daunting task. In *quick* mode we observe 6X to 9X speedup while scaling up to hundreds of datasets. Raspberry, an in-house tool was developed for quality control of raw data is integrated into the pipeline.

## Materials and Methods

Raspberry, an in-house tool to process large datasets generated from Illumina next generation sequencers was developed in C language using htslib C library (http://htslib.org) API (Application Programming Interface). This library was chosen for efficiency in processing datasets with low memory consumption and faster processing. In addition, it supports reading compressed or uncompressed datasets. OpenMP (http://openmp.org) (v4.0) was used to process datasets in batches. Cmake (http://www.cmake.org) (v2.8) build system was used to develop Raspberry as it supports cross-platform compilation. Static binaries compiled on x86_64 machine architecture are provided with the software to facilitate direct use of the application by naive users.

The NGS-QCbox pipeline was implemented by integrating NGS tools such as Bpipe, Sickle, bedtools, Bowtie 2, SAMtools and in-house software, Raspberry. Python, C, Bash shell was used extensively in building the application. Parallelization was envisaged at different levels. Python’s multiprocessing module was used to implement batch processing based on the number of processors requested by the user. Bpipe’s inbuilt parallelization of task blocks feature was used towards computing genome coverage and variation information simultaneously.

For benchmarking, a dataset of size 4.38 Gb, comprising of 100 bp paired-end reads were simulated from chickpea genome [5] using ART simulator [16] (v2.1.8) (http://www.niehs.nih.gov/research/resources/software/biostatistics/art/) at 10 fold genome coverage. The dataset is publicly available (https://github.com/CEG-ICRISAT/NGS-QCbox/blob/master/README.md#datasets-used-for-testing) on iPlant resource [17] (www.iplantcollaborative.org). The test was conducted in quick mode on an Ubuntu Linux server with x86_64 architecture.

This was used to evaluate the performance of NGS-QCbox against five similar tools such as Prinseq-lite, NGS QC Toolkit, HTSeq, FastQC and FastX. To establish a proof of concept towards scalability, sample sets of 100, 200 and 300 samples were drawn from the same dataset to test the performance of the tools with one processor and 20 processors (in parallel) independently. This enables comparison of runtime in serial versus parallel modes of NGS-QCbox. All the benchmarking tests were performed on the 64-bit server with no load.

## Supporting Information

S1 Table Sample output of the analysis of simulated NGS data using NGS-QCbox pipeline. (XLS)

## References

[1] Thudi M, Li Y, Jackson SA, May GD, Varshney RK. Current state-of-art of sequencing technologies for plant genomics research. Briefings in Functional Genomics. 2012;11(1):3 **–** 11. 10.1093/bfgp/elr045 22345601

[2] McCouch S, Baute GJ, Bradeen J, Bramel P, Bretting PK, Buckler E, et al Agriculture: feeding the future. Nature. 2013;499(7456):23 **–** 24. 10.1038/499023a 23823779

[3] Jiao Y, Zhao H, Ren L, Song W, Zeng B, Guo J, et al Genome-wide genetic changes during modern breeding of maize. Nature genetics. 2012;44(7):812 **–** 815. 10.1038/ng.2312 22660547

[4] Mace ES, Tai S, Gilding EK, Li Y, Prentis PJ, Bian L, et al Whole-genome sequencing reveals untapped genetic potential in Africa's indigenous cereal crop sorghum. Nature communications. 2013;4 10.1038/ncomms3320 23982223 PMC3759062

[5] Varshney RK, Song C, Saxena RK, Azam S, Yu S, Sharpe AG, et al Draft genome sequence of chickpea (*Cicer arietinum*) provides a resource for trait improvement. Nature biotechnology. 2013;31(3):240 **–** 246. 10.1038/nbt.2491 23354103

[6] Gardner SN, Hall BG. When whole-genome alignments just won't work: kSNP v2 software for alignment-free SNP discovery and phylogenetics of hundreds of microbial genomes. PLoS One. 2013;8(12):e81760 10.1371/journal.pone.0081760 24349125 PMC3857212

[7] Bertels F, Silander OK, Pachkov M, Rainey PB, van Nimwegen E. Automated reconstruction of whole-genome phylogenies from short-sequence reads. Molecular biology and evolution. 2014;31(5):1077 **–** 1088. 10.1093/molbev/msu088 24600054 PMC3995342

[8] Patel RK, Jain M. NGS QC Toolkit: a toolkit for quality control of next generation sequencing data. PloS one. 2012;7(2):e30619 10.1371/journal.pone.0030619 22312429 PMC3270013

[9] Anders S, Pyl PT, Huber W. HTSeq—A Python framework to work with high-throughput sequencing data. Bioinformatics. 2014; btu638. 10.1093/bioinformatics/btu638 PMC4287950 25260700

[10] Cock PJ, Fields CJ, Goto N, Heuer ML, Rice PM. The Sanger FASTQ file format for sequences with quality scores, and the Solexa/Illumina FASTQ variants. Nucleic acids research. 2010;38(6):1767–1771. 10.1093/nar/gkp1137 20015970 PMC2847217

[11] Sadedin SP, Pope B, Oshlack A. Bpipe: a tool for running and managing bioinformatics pipelines. Bioinformatics. 2012;28(11):1525 **–** 1526. 10.1093/bioinformatics/bts167 22500002

[12] Langmead B, Salzberg SL. Fast gapped-read alignment with Bowtie 2. Nature methods. 2012;9(4):357 **–** 359. 10.1038/nmeth.1923 22388286 PMC3322381

[13] Li H, Handsaker B, Wysoker A, Fennell T, Ruan J, Homer N, et al The sequence alignment/map format and SAMtools. Bioinformatics. 2009;25(16):2078 **–** 2079. 10.1093/bioinformatics/btp352 19505943 PMC2723002

[14] Quinlan AR, Hall IM. BEDTools: a flexible suite of utilities for comparing genomic features. Bioinformatics. 2010;26(6):841 **–** 842. 10.1093/bioinformatics/btq033 20110278 PMC2832824

[15] Schmieder R, Edwards R. Quality control and preprocessing of metagenomic datasets. Bioinformatics. 2011;27(6):863 **–** 864 10.1093/bioinformatics/btr026 21278185 PMC3051327

[16] Huang W, Li L, Myers JR, Marth GT. ART: a next-generation sequencing read simulator. Bioinformatics. 2012;28(4):593–594. 10.1093/bioinformatics/btr708 22199392 PMC3278762

[17] Goff SA, Vaughn M, McKay S, Lyons E, Stapleton AE, Gessler D, et al The iPlant collaborative: cyberinfrastructure for plant biology. Frontiers in plant science. 2011;2 10.3389/fpls.2011.00034 22645531 PMC3355756

